# Supplementary material for: Modeling glioblastoma heterogeneity as a dynamic network of cell states
Source: Mol Syst Biol. 2021 Sep 16;17(9):e10105. doi: 10.15252/msb.202010105 (PMC8444284; doi:10.15252/msb.202010105)
Supplement: Supplementary file 5 — Source Data for Figure 3 [file MSB-17-e10105-s001.zip › Figure3A_sourcedata/GSEA_3065/hallmarks_state2.GseaPreranked.1623416269049/HALLMARK_HYPOXIA.html]

Details for gene set HALLMARK\_HYPOXIA[GSEA]

|  || Dataset | state2 |
| Phenotype | NoPhenotypeAvailable |
| Upregulated in class | na\_neg |
| GeneSet | HALLMARK\_HYPOXIA |
| Enrichment Score (ES) | -0.5145595 |
| Normalized Enrichment Score (NES) | -2.0569015 |
| Nominal p-value | 0.0 |
| FDR q-value | 0.0 |
| FWER p-Value | 0.0 |
Table: GSEA Results Summary

  

Fig 1: Enrichment plot: HALLMARK\_HYPOXIA      
 Profile of the Running ES Score & Positions of GeneSet Members on the Rank Ordered List

  

| PROBE | GENE SYMBOL | GENE\_TITLE | RANK IN GENE LIST | RANK METRIC SCORE | RUNNING ES | CORE ENRICHMENT || 1 | SAP30 |  |  | 164 | 0.303 | 0.0087 | No |
| 2 | TPI1 |  |  | 301 | 0.231 | 0.0141 | No |
| 3 | LDHA |  |  | 506 | 0.179 | 0.0078 | No |
| 4 | ENO1 |  |  | 717 | 0.148 | -0.0019 | No |
| 5 | PGK1 |  |  | 726 | 0.146 | 0.0100 | No |
| 6 | MT2A |  |  | 731 | 0.146 | 0.0223 | No |
| 7 | CSRP2 |  |  | 847 | 0.133 | 0.0215 | No |
| 8 | UGP2 |  |  | 851 | 0.132 | 0.0326 | No |
| 9 | GAPDH |  |  | 933 | 0.125 | 0.0348 | No |
| 10 | HDLBP |  |  | 1436 | 0.090 | -0.0113 | No |
| 11 | TPD52 |  |  | 1493 | 0.087 | -0.0098 | No |
| 12 | GRHPR |  |  | 1610 | 0.081 | -0.0152 | No |
| 13 | PRDX5 |  |  | 1642 | 0.080 | -0.0115 | No |
| 14 | JUN |  |  | 1805 | 0.072 | -0.0227 | No |
| 15 | DPYSL4 |  |  | 1860 | 0.070 | -0.0224 | No |
| 16 | MAP3K1 |  |  | 2140 | 0.060 | -0.0472 | No |
| 17 | SLC25A1 |  |  | 2256 | 0.055 | -0.0547 | No |
| 18 | GPI |  |  | 2330 | 0.053 | -0.0580 | No |
| 19 | PGM2 |  |  | 2339 | 0.052 | -0.0543 | No |
| 20 | PFKL |  |  | 2489 | 0.048 | -0.0661 | No |
| 21 | BCL2 |  |  | 2499 | 0.048 | -0.0629 | No |
| 22 | MIF |  |  | 2504 | 0.047 | -0.0593 | No |
| 23 | JMJD6 |  |  | 2521 | 0.047 | -0.0569 | No |
| 24 | SIAH2 |  |  | 2599 | 0.045 | -0.0613 | No |
| 25 | NAGK |  |  | 2611 | 0.044 | -0.0586 | No |
| 26 | ETS1 |  |  | 2998 | 0.034 | -0.0972 | No |
| 27 | ENO2 |  |  | 3006 | 0.034 | -0.0950 | No |
| 28 | VHL |  |  | 3007 | 0.034 | -0.0921 | No |
| 29 | PFKP |  |  | 3170 | 0.030 | -0.1068 | No |
| 30 | CASP6 |  |  | 3357 | 0.027 | -0.1245 | No |
| 31 | RBPJ |  |  | 3541 | 0.023 | -0.1421 | No |
| 32 | ISG20 |  |  | 3567 | 0.023 | -0.1429 | No |
| 33 | PGM1 |  |  | 3936 | 0.016 | -0.1810 | No |
| 34 | SLC37A4 |  |  | 3957 | 0.016 | -0.1817 | No |
| 35 | GALK1 |  |  | 4003 | 0.015 | -0.1852 | No |
| 36 | CDKN1B |  |  | 4019 | 0.015 | -0.1855 | No |
| 37 | RRAGD |  |  | 4059 | 0.015 | -0.1884 | No |
| 38 | EXT1 |  |  | 4083 | 0.014 | -0.1897 | No |
| 39 | NFIL3 |  |  | 4213 | 0.012 | -0.2025 | No |
| 40 | ALDOA |  |  | 4435 | 0.008 | -0.2255 | No |
| 41 | S100A4 |  |  | 4483 | 0.008 | -0.2299 | No |
| 42 | PDK3 |  |  | 4547 | 0.007 | -0.2361 | No |
| 43 | FAM162A |  |  | 4622 | 0.005 | -0.2436 | No |
| 44 | XPNPEP1 |  |  | 4637 | 0.005 | -0.2446 | No |
| 45 | DUSP1 |  |  | 4653 | 0.005 | -0.2458 | No |
| 46 | VEGFA |  |  | 4721 | 0.004 | -0.2527 | No |
| 47 | PLIN2 |  |  | 4837 | 0.002 | -0.2649 | No |
| 48 | KDM3A |  |  | 5222 | -0.004 | -0.3058 | No |
| 49 | ERO1A |  |  | 5380 | -0.006 | -0.3221 | No |
| 50 | BNIP3L |  |  | 5447 | -0.008 | -0.3285 | No |
| 51 | B3GALT6 |  |  | 5563 | -0.009 | -0.3401 | No |
| 52 | LARGE1 |  |  | 5611 | -0.010 | -0.3442 | No |
| 53 | HS3ST1 |  |  | 5801 | -0.013 | -0.3634 | No |
| 54 | HK1 |  |  | 5827 | -0.014 | -0.3649 | No |
| 55 | HOXB9 |  |  | 5998 | -0.017 | -0.3817 | No |
| 56 | CAVIN3 |  |  | 6155 | -0.019 | -0.3968 | No |
| 57 | ATP7A |  |  | 6343 | -0.022 | -0.4149 | No |
| 58 | DDIT3 |  |  | 6433 | -0.024 | -0.4224 | No |
| 59 | ERRFI1 |  |  | 6445 | -0.024 | -0.4214 | No |
| 60 | AKAP12 |  |  | 6471 | -0.025 | -0.4220 | No |
| 61 | ADORA2B |  |  | 6618 | -0.028 | -0.4352 | No |
| 62 | ATF3 |  |  | 6737 | -0.030 | -0.4453 | No |
| 63 | SCARB1 |  |  | 6752 | -0.031 | -0.4441 | No |
| 64 | NR3C1 |  |  | 6802 | -0.032 | -0.4467 | No |
| 65 | GBE1 |  |  | 6821 | -0.032 | -0.4458 | No |
| 66 | PLAUR |  |  | 6906 | -0.034 | -0.4518 | No |
| 67 | PPP1R15A |  |  | 7004 | -0.037 | -0.4591 | No |
| 68 | KLF6 |  |  | 7010 | -0.037 | -0.4564 | No |
| 69 | NDST1 |  |  | 7077 | -0.038 | -0.4602 | No |
| 70 | FOS |  |  | 7222 | -0.043 | -0.4719 | No |
| 71 | TPST2 |  |  | 7315 | -0.045 | -0.4779 | No |
| 72 | KDELR3 |  |  | 7339 | -0.046 | -0.4764 | No |
| 73 | CCNG2 |  |  | 7353 | -0.046 | -0.4738 | No |
| 74 | CDKN1C |  |  | 7524 | -0.051 | -0.4876 | No |
| 75 | NDRG1 |  |  | 7538 | -0.051 | -0.4845 | No |
| 76 | MXI1 |  |  | 7589 | -0.053 | -0.4853 | No |
| 77 | F3 |  |  | 7662 | -0.055 | -0.4883 | No |
| 78 | FOSL2 |  |  | 7698 | -0.056 | -0.4871 | No |
| 79 | ILVBL |  |  | 7855 | -0.061 | -0.4986 | No |
| 80 | CA12 |  |  | 8005 | -0.067 | -0.5087 | Yes |
| 81 | CHST3 |  |  | 8008 | -0.068 | -0.5030 | Yes |
| 82 | NEDD4L |  |  | 8042 | -0.069 | -0.5006 | Yes |
| 83 | TIPARP |  |  | 8047 | -0.069 | -0.4951 | Yes |
| 84 | GAA |  |  | 8060 | -0.069 | -0.4903 | Yes |
| 85 | SDC3 |  |  | 8064 | -0.070 | -0.4846 | Yes |
| 86 | EGFR |  |  | 8094 | -0.071 | -0.4815 | Yes |
| 87 | GLRX |  |  | 8100 | -0.071 | -0.4759 | Yes |
| 88 | BTG1 |  |  | 8156 | -0.075 | -0.4753 | Yes |
| 89 | SLC6A6 |  |  | 8167 | -0.075 | -0.4698 | Yes |
| 90 | SDC4 |  |  | 8172 | -0.076 | -0.4637 | Yes |
| 91 | VLDLR |  |  | 8186 | -0.077 | -0.4584 | Yes |
| 92 | TMEM45A |  |  | 8214 | -0.078 | -0.4546 | Yes |
| 93 | CITED2 |  |  | 8322 | -0.084 | -0.4588 | Yes |
| 94 | P4HA1 |  |  | 8398 | -0.089 | -0.4591 | Yes |
| 95 | SLC2A3 |  |  | 8419 | -0.090 | -0.4534 | Yes |
| 96 | ANKZF1 |  |  | 8480 | -0.094 | -0.4517 | Yes |
| 97 | KLHL24 |  |  | 8545 | -0.100 | -0.4499 | Yes |
| 98 | ZNF292 |  |  | 8563 | -0.101 | -0.4430 | Yes |
| 99 | GPC4 |  |  | 8568 | -0.101 | -0.4346 | Yes |
| 100 | DDIT4 |  |  | 8601 | -0.103 | -0.4290 | Yes |
| 101 | FOXO3 |  |  | 8616 | -0.104 | -0.4215 | Yes |
| 102 | CHST2 |  |  | 8670 | -0.109 | -0.4178 | Yes |
| 103 | P4HA2 |  |  | 8700 | -0.111 | -0.4112 | Yes |
| 104 | IRS2 |  |  | 8704 | -0.111 | -0.4019 | Yes |
| 105 | TGFBI |  |  | 8761 | -0.117 | -0.3977 | Yes |
| 106 | MYH9 |  |  | 8778 | -0.119 | -0.3891 | Yes |
| 107 | BHLHE40 |  |  | 8804 | -0.123 | -0.3811 | Yes |
| 108 | DTNA |  |  | 8828 | -0.126 | -0.3726 | Yes |
| 109 | TPBG |  |  | 8829 | -0.127 | -0.3616 | Yes |
| 110 | IDS |  |  | 8874 | -0.133 | -0.3548 | Yes |
| 111 | ANXA2 |  |  | 8891 | -0.134 | -0.3449 | Yes |
| 112 | SDC2 |  |  | 9002 | -0.149 | -0.3438 | Yes |
| 113 | CAVIN1 |  |  | 9071 | -0.161 | -0.3371 | Yes |
| 114 | PRKCA |  |  | 9072 | -0.161 | -0.3231 | Yes |
| 115 | HMOX1 |  |  | 9084 | -0.163 | -0.3101 | Yes |
| 116 | HEXA |  |  | 9129 | -0.172 | -0.2999 | Yes |
| 117 | CAV1 |  |  | 9130 | -0.172 | -0.2849 | Yes |
| 118 | COL5A1 |  |  | 9148 | -0.177 | -0.2714 | Yes |
| 119 | SRPX |  |  | 9161 | -0.179 | -0.2571 | Yes |
| 120 | PAM |  |  | 9280 | -0.224 | -0.2503 | Yes |
| 121 | PNRC1 |  |  | 9294 | -0.229 | -0.2319 | Yes |
| 122 | CDKN1A |  |  | 9301 | -0.230 | -0.2125 | Yes |
| 123 | GPC1 |  |  | 9326 | -0.250 | -0.1934 | Yes |
| 124 | CXCR4 |  |  | 9328 | -0.251 | -0.1717 | Yes |
| 125 | SLC2A1 |  |  | 9349 | -0.272 | -0.1503 | Yes |
| 126 | BCAN |  |  | 9353 | -0.276 | -0.1266 | Yes |
| 127 | STC1 |  |  | 9378 | -0.314 | -0.1020 | Yes |
| 128 | WSB1 |  |  | 9393 | -0.350 | -0.0731 | Yes |
| 129 | HSPA5 |  |  | 9406 | -0.395 | -0.0400 | Yes |
| 130 | IGFBP3 |  |  | 9424 | -0.502 | 0.0017 | Yes |
Table: GSEA details [plain text format]

  

Fig 2: HALLMARK\_HYPOXIA: Random ES distribution      
 Gene set null distribution of ES for **HALLMARK\_HYPOXIA**
